# Biodegradation of crude oil by immobilized *Exiguobacterium* sp. AO-11 and shelf life evaluation

**DOI:** 10.1038/s41598-021-92122-1

**Published:** 2021-06-21

**Authors:** Chatsuda Sakdapetsiri, Nitchakarn Kaokhum, Onruthai Pinyakong

**Affiliations:** 1 Microbial Technology for Marine Pollution Treatment Research Unit, Department of Microbiology, Faculty of Science, Chulalongkorn University, Bangkok, 10330 Thailand; 2 Research Program on Remediation Technologies for Petroleum Contamination, Center of Excellence on Hazardous Substance Management (HSM), Chulalongkorn University, Bangkok, Thailand

**Keywords:** Microbiology, Environmental sciences

## Abstract

*Exiguobacterium* sp. AO-11 was immobilized on bio-cord at 10^9^ CFU g^−1^ carrier for the removal of crude oil from marine environments. To prepare a ready-to-use bioremediation product, the shelf life of the immobilized cells was calculated. Approximately 90% of 0.25% (v/v) crude oil removal was achieved within 9 days when the starved state of immobilized cells was used. The oil removal activity of the immobilized cells was maintained in the presence of oil dispersant (89%) and at pH values of 7–9. Meanwhile, pH, oil concentration and salinity affected the oil removal efficacy. The immobilized cells could be reused for at least 5 cycles. The Arrhenius equation describing the relationship between the rate of reaction and temperature was validated as a useful model of the kinetics of retention of activity by an immobilized biocatalyst. It was estimated that the immobilized cells could be stored in a non-vacuum bag containing phosphate buffer (pH 7.0) at 30 °C for 39 days to retain the cells at 10^7^ CFU g^−1^ carrier and more than 50% degradation activity. These results indicated the potential of using bio-cord-immobilized crude oil-degrading *Exiguobacterium* sp. AO-11 as a bioremediation product in a marine environment.

## Introduction

Crude oil contamination in a marine environment causes serious damage to aquatic life, human health and the ecosystem^1^. According to the oil spill response protocol of the United States Environmental Protection Agency (USEPA), physical and chemical techniques using oil spill booms and oil dispersants, respectively, have been used as quick solutions to treat oil contamination^2^. Biological treatment was subsequently employed to remove the remaining oil residues to reduce the long-term health and ecological effects^3^. Hence, bioaugmentation using oil-degrading bacteria immobilized on oil-adsorbing material often offers efficient oil removal due to both its physical and biological activities^4^. Immobilized cells exhibiting high biological activity in the presence of oil dispersant are required. Reusable immobilized cells are considered to be a promising biological treatment that can be combined with physical and chemical treatments for better sustainability, self-stimulation and environmental friendliness^5^.

Non-pathogenic *Exiguobacterium* sp. AO-11 capable of degrading crude oil was recently isolated and evaluated for its ability to remove crude oil from sea sediment microcosms^6^. This bacterium was also developed as a liquid ready-to-use inoculum. However, the free cell form may not be applicable for the bioremediation of oil contamination in seawater systems due to uncontrollable factors, such as the effect of dilution in an open-water system, and the variation of environmental factors (pH, salinity and temperature) resulted in a substantial decline in the efficiency of remediation and loss of effective strains^7^.

Thus, to enhance bioremediation efficacy, immobilized microbial technology, which offers several advantages, such as high microbial density, stable biological activity, less biomass loss, resistance to toxic chemicals and strong environmental tolerance^8^, is worth developing. Immobilization based on cell attachment on a supporting material requires the biofilm-producing property of bacteria to facilitate cell adherence, resulting in stable cell immobilization^9^. A suitable carrier is one of the important factors related to the efficiency of crude oil removal. The carrier should be nontoxic, low-cost, and easily separated from the environment^10^. Additionally, the carrier should have a high cell mass loading, oil adsorption capacity, and a long shelf life. A variety of support materials, including alginate, bamboo charcoal, bagasse, cotton fibers, chitin, and chitosan flakes, have been used to immobilize bacteria for long periods of time^11–18^. Nevertheless, they are difficult to prepare, have low crude oil adsorption capacity, and are difficult to recover from the environment, hence making them impractical for commercial use. Therefore, inert support materials are suitable immobilization carriers. Such materials not only have high oil adsorption properties and are easy to use but also reduce the cost of regeneration and recirculation and minimize bacterial loss^13^.

The major difficulty in the commercialization of bioremediation products is the development of a shelf-stable formulated product that retains the efficacy and viability of cells^19^. The shelf life is commonly estimated by accelerated stability tests, in which the formulated product is stored under different stress conditions, such as temperature and pH. The recommended storage conditions can be predicted using relationships between the acceleration factor and the death rate constant and are characterized by the Arrhenius equation^20^. The data obtained under such accelerated tests are used to predict real-time storage behavior via extrapolation^21^.

Several studies have used immobilization technology for the bioremediation of crude oil-contaminated marine environments^5,12,22,23^. Further, a literature review shows that significant work has been done by various researchers on the selection of immobilized carriers, factors affecting crude oil removal ability, and the reusability of immobilized cells. However, very few reports are available on the crude oil removal efficacy of *Exiguobacterium* species in terms of the effects of starvation on crude oil remediation and shelf-life evaluation for long-term storage, which are not fully understood, although this information is important for product development. Therefore, this study introduces an immobilized bioremediation technology using *Exiguobacterium* sp. AO-11 immobilized on polypropylene fine fibers, or bio-cord, which is inexpensive, can carry a high volume of immobilized cells and has crude oil adsorption capacity. Moreover, the effect of cell starvation is a key strategy for managing stress conditions that affect biodegradation activity. The influence of abiotic factors that impacted crude oil removal efficacy, including the concentration of crude oil, pH, salinity, and the presence of oil dispersant, by immobilized cells was evaluated. Finally, accelerated storage testing was performed to determine the death rate constant of the immobilized product stored at different temperatures according to the Arrhenius equation. A predicted shelf life equation of immobilized *Exiguobacterium* sp. AO-11 was generated and validated from the number of viable cells after storage. The results from this research will provide a further theoretical basis and technical support for the development of bioremediation products for oil-polluted marine environments.

## Materials and methods

### Chemicals and cultivation media

Mixed crude oil of Arabian light and Arabian extra light was generously supplied by Thai Oil Public Company Limited (Thailand). For the removal of crude oil, strain AO-11 was cultivated in modified nutrient seawater (MNSW, pH 7.0 ± 0.2, salinity 32 per mille (‰)) broth containing the following compounds (per liter): 1.0 g of NH_4_NO_3_, 0.02 g of K_2_HPO_4_, 0.02 g of C_6_H_5_FeO_7_, and 0.5 g of yeast extract dissolved in 1,000 mL of filtered seawater. Inoculum preparation and bacterial immobilization on the carrier were performed in 0.1 × Zobell marine medium (Himedia, India) (pH 7.0 ± 0.2, salinity 10 ‰). The sonication buffer was phosphate urea magnesium sulfate (PUM) buffer^24^ consisting of the following (per liter): 16.9 g of K_2_HPO_4_, 7.3 g of KH_2_PO_4_, 1.8 g of urea, and 0.2 g of MgSO_4_∙7H_2_O. The dispersant, Slickgone NS (Dasic, England), containing 1–10% (w/w) anionic surfactant and > 50% (w/w) kerosene, was generously supplied by Thai Oil PCL Company. All reagents were of analytical grade.

### Microorganisms and inoculum preparation

The petroleum hydrocarbon-degrading bacterium *Exiguobacterium* sp. AO-11 (MSCU 0807), previously isolated from the sediment of Phrao Bay (Ao Phrao), Rayong Province, Thailand^6^, was used in this study. To prepare the inoculum, strain AO-11 was cultivated in 100 mL of 0.1 × Zobell medium contained in 250-mL Erlenmeyer flasks at room temperature (30–33 °C) with a shaking speed of 200 rpm for 16–18 h. Cells were harvested by centrifugation at 8,000 rpm for 10 min at 4 °C and were then washed twice with 0.85% (w/v) NaCl solution. The cells were then resuspended in 0.85% (w/v) NaCl solution, and the optical density was adjusted at 600 nm to 1 by a spectrophotometer (Shimadzu, Japan), which was equivalent to 10^8^ CFU mL^−1^.

### Quantification of biofilm formation by *Exiguobacterium* sp. AO-11

Biofilm formation was evaluated according to the method of Stepanović, et al.^25^. Briefly, the inoculum of AO-11 at a concentration of approximately 10^8^ CFU mL^−1^ underwent a serial tenfold dilution in MNSW and was seeded in 96-well polystyrene tissue culture-treated plates (Falcon, USA). Uninoculated wells containing sterile MNSW, considered to be the negative controls, were used as blanks. The experiment was incubated at room temperature (30–33 °C) for 3 and 7 days. After incubation, suspended cells were discarded, and the wells were gently washed with sterile distilled water three times. To stain biofilms, a solution of 0.1% (w/v) crystal violet in water was added to each well of the microtiter plate and incubated for 10–15 min. Subsequently, stained wells were rinsed 3–4 times with water, and then the microtiter plate was turned upside down and dried for a few hours or overnight. To evaluate biofilm formation, 125 µL of 30% (v/v) acetic acid in water was added into each stained well of the microtiter plate to solubilize the crystal violet and incubated for 10–15 min. Then, the solubilized crystal violet was transferred to a flat-bottomed microtiter dish, and absorbance at 540 nm was measured using a plate reader (model 2030 multilabel reader; PerkinElmer, Finland) and with 30% (v/v) acetic acid in water as the blank. All experiments were performed in triplicate. The results were categorized as no biofilm formation and weak, moderate, and strong biofilm formation compared to the OD control (ODc) according to published criteria by Rampacci et al.^26^.

### Examination of the oil adsorption capacity and number of bacteria attached to carriers

The various carriers, polyurethane foam (PUF) (1 × 1 × 1 cm)^15^ (Thai Foam Product Co., Ltd, Thailand), aquaporous gel (1 × 1 × 1 cm) (Nisshinbo Chemical Inc., Japan) and bio-cord (0.2 × 2 cm) (TBR Co., Ltd, Japan), were used as candidates for immobilizing strain AO-11. To determine the maximum oil adsorption capacity, 200 mg of each carrier was placed in a 125-mL Erlenmeyer flask containing 25 mL of seawater followed by shaking at 200 rpm for 1 h. Then, 100 µl (equivalent 76 mg) of crude oil was gradually added in each experiment, followed by shaking at 200 rpm for 1 h. Crude oil was repeatedly added in each experiment until an oil slick was observed. The volume of crude oil added in each experiment was recorded. All experiments were performed in triplicate. The adsorption capacity was calculated as follows: [(Adsorbed oil/dry weight of carrier)].

Bacterial immobilization onto the carriers mentioned above was performed, and the number of attached bacteria on each carrier was evaluated. One gram of each carrier was soaked in 90 mL of 0.1 × Zobell marine broth contained in 250-mL conical flasks and inoculated with 10% (v/v) inoculum of strain AO-11. The experiments were incubated at room temperature (30–33 °C) on a rotary shaker at 200 rpm for 3 days. To evaluate the viability of cells attached to the carrier, the suspension was discarded, and immobilized carriers were rinsed with 0.85% (w/v) NaCl three times to remove unattached cells. The immobilized carrier was resuspended in 100 mL of PUM buffer. Bacterial extraction was performed by sonication in a sonication bath for 2 min with two cycle of vigorous shaking to release cells from the carrier. The cells were centrifuged at 8,000 rpm and 4 °C for 15 min and resuspended in 1 mL of 0.85% (w/v) NaCl. The viable cell count was determined following the standard drop plate technique^27^ on Zobell marine agar. All experiments were performed in triplicate. The carrier with the highest capacity for both crude oil adsorption and bacterial attachment to the carrier was chosen for subsequent experiments.

### Determination of the crude oil removal activity by free and immobilized bacteria

Crude oil removal analysis was conducted in MNSW medium (pH 7.0 ± 0.2, salinity 32‰) supplemented with 0.25% (v/v) crude oil (approximately 2000 ppm). In the experiment involving free cells, the inoculum was added to give a final concentration of 10^7^ CFU mL^−1^ in 100 mL of MNSW contained in a 250-mL Erlenmeyer flask. The cultured flasks were incubated at room temperature (30–33 °C) and shaken in a rotary shaker at 200 rpm for 9 days. For immobilized AO-11 cells, 1 g of immobilized AO-11 cells, which was equivalent to 10^9^ CFU g^−1^ carrier, was added to the same culture medium and incubated under the same conditions. The initial cell number in both experiments was 10^9^ CFU treatment^−1^. To quantify abiotic losses, especially due to evaporation, control experiments using only medium or selected carriers and supplemented with 0.25% (v/v) crude oil without bacterial cells were set up. All experiments were performed in triplicate. The crude oil residue was extracted with hexane following the method reported by Nopcharoenkul, et al.^9^. The oil removal efficiency of free cells compared with immobilized AO-11 on day 9 was determined using gas chromatography–flame ionization detector (GC-FID, Agilent Model 6890, Agilent Technologies, Palo Alto, CA, USA) equipped with an HP-5 column (30 m × 0.25 mm × 0.25 µm). The detector temperature was set at 320 °C and was operated in splitless mode. The conditions used were as follows: a 2-min hold at 40 °C and ramp-up from 40 to 320 °C at 10 °C min^−1^. Abiotic losses during crude oil degradation were calculated based on chromatographic peak areas as follows:

$${\text{Abiotic}}\;{\text{control~}}\left( {\text{\% }} \right) = \left( {\frac{{C_{0} - C_{9} }}{{C_{0} }}} \right) \times 100$$

where C_0_ is the amount in the control on day 0 and C_9_ is the residual amount in the control on day 9.

To calculate the removal percentage (%) by free cells or immobilized AO-11 cells without the effect of abiotic degradation, the removal percentage (%) was calculated based on chromatographic peak areas as follows:

$${\text{Removal}}\;{\text{percentage~}}\left( {\text{\% }} \right) = \left( {\frac{{C - Exp}}{C}} \right) \times 100$$

where C is the amount in the control experiment without bacteria on day 9 and Exp is the residual amount in the experiment on day 9.

### Factors affecting crude oil removal by immobilized *Exiguobacterium* sp. AO-11

#### Effect of the crude oil concentration

The effect of the crude oil concentration on the removal efficacy was studied using 0.125, 0.25, 0.5, 1.0 and 1.5% (v/v) crude oil. One gram of immobilized cells was added into a 250-mL Erlenmeyer flask containing 100 mL of MNSW medium (pH 7.0 ± 0.2, salinity 32‰). The flasks containing sterilized carrier and each crude oil concentration were set as control experiments. All experiments were incubated at room temperature (30–33 °C) and shaken in a rotary shaker at 200 rpm for 9 days. All experiments were performed in triplicate. Residual crude oil was extracted by hexane, and the crude oil removal efficiency was determined as described in previous section.

#### Effect of the initial pH

The pH of MNSW medium (initial pH 7.0 ± 0.2, salinity 32 ‰) was adjusted to 6.0, 7.0, 8.0 and 9.0 using 1 mol L^−1^ HCl or 1 M NaOH solutions. Immobilized cells (1 g) were added into a 250-mL Erlenmeyer flask containing 100 mL of pH-adjusted MNSW medium. The experiments were conducted with 0.25% (v/v) crude oil. Flasks containing sterilized carriers were used as a control experiment. All experiments were performed in triplicate and incubated at room temperature (30–33 °C) in a rotary shaker at 200 rpm for 9 days. Crude oil residue was extracted by hexane, and the crude oil removal efficiency was determined as described in previous section.

#### Effect of salinity

To determine the effect of salinity, MNSW (initial pH 7.0 ± 0.2) was prepared by adding distilled water instead of seawater, and the salinity was adjusted to the desired concentration (0, 22, 32, and 42 ‰) by adding sea salts (Sigma-Aldrich, Germany) to MNSW. Immobilized cells (1 g) were added into a 250-mL Erlenmeyer flask containing 100 mL of MNSW medium and 0.25% (v/v) crude oil. Flasks containing sterilized carriers were used as a control experiment. All experiments were performed in triplicate and incubated at room temperature (30–33 °C) in a rotary shaker at 200 rpm for 9 days. Residual crude oil was extracted by hexane, and the crude oil removal efficiency was determined as described in previous section.

#### Effect of starvation

Inocula of free and immobilized AO-11 cells were prepared as described above. To starve free cells, free cell inoculum was centrifuged at 8,000 rpm and 4 °C for 20 min. Cell pellets were washed twice with sterile distilled water and resuspended in 0.85% (w/v) NaCl solution. Immobilized AO-11 cells were washed twice in sterile distilled water and resuspended in 0.85% (w/v) NaCl solution. Bacterial starvation was performed by continuous shaking of the cell suspension or immobilized cells at 200 rpm for 0, 24, 48, 72, and 96 h at room temperature (30–33 °C). The experiments were performed in triplicate. After starvation, the crude oil removal ability was examined as described in previous section. The morphologies of both free and immobilized cells were observed by scanning electron microscopy (SEM).

#### Effect of oil dispersant

The dispersant, Slickgone NS, was added to achieve a dispersant-to-oil-ratio (DOR) of approximately 1:25 in both free and immobilized cell experiments after starvation by continuous shaking of cell pellets or immobilized cells in 0.85% (w/v) NaCl solution at 200 rpm for 96 and 72 h, respectively, at room temperature (30–33 °C). To determine cell viability after exposure to Slickgone NS, viable cell count was determined following the standard drop plate technique^27^ on Zobell marine agar. Furthermore, the degradation efficiency after exposure to Slickgone NS was analyzed as described in previous section. All experiments were performed in triplicate.

### Reusability of immobilized *Exiguobacterium* sp. AO-11 cells

To examine reusability, immobilized AO-11 cells were incubated in 100 mL of MNSW (pH 7.0 ± 0.2, salinity 32‰) containing 0.25% (v/v) crude oil. The experiments were shaken at 200 rpm on a rotary shaker for 9 days/cycle at room temperature. After each incubation cycle, the immobilized AO-11 cells were washed with phosphate buffer (pH 7.0) and resuspended in 100 mL of fresh MNSW medium (pH 7.0 ± 0.2, salinity 32‰) containing 0.25% (v/v) crude oil. The immobilized cells were reused under the same operating conditions for up to 5 cycles. MNSW medium supplemented with 0.25% (v/v) crude oil but without immobilized AO-11 cells served as the control experiment. All experiments were performed in triplicate. The crude oil removal efficiency was determined as described above. The SEM of immobilized cells was analyzed after 1, 3, and 5 cycles of use.

### Storage of immobilized *Exiguobacterium* sp. AO-11 product

#### Protective agent selection

To assess the effect of adding each protective agent on the storage of immobilized cells, individual protective agents at a concentration of 2% (w/v) for trehalose and sucrose and 1% (w/v) for polyvinylpyrrolidone (PVP), glycerol and polyethylene glycol 6000 (PEG 6000) were prepared in phosphate buffer (PB). The experiments were examined by adding 5 mL of solution to a 0.08-mm-thick nylon PE bag (15 × 10 cm) containing 1 g of immobilized cells. The bags were sealed with a kitchen-use sealer (Spring Green Evolution, Thailand) under either vacuum by the evacuation of air for five seconds (with the vacuum level up to − 0.08 mPa) or non-vacuum conditions and were stored at 4 and 30 °C. Immobilized cells added to phosphate buffer without protective agent served as the control experiment. The samples were collected at days 15 and 30 of storage. All experiments were performed in triplicate. Bacterial survival was evaluated by the drop plate technique^27^, and the percent survival was calculated as described by Nopcharoenkul et al.^28^.

#### Accelerated test at different storage temperatures

To estimate the shelf life of immobilized AO-11, 1 g of immobilized cells was placed in a 0.08-mm-thick nylon PE bag (15 × 10 cm) with the addition of 5 mL of phosphate buffer. After that, the bag was sealed with a kitchen-use sealer under non-vacuum conditions. The immobilized products were maintained at different temperatures (5, 15, 30, and 40 °C). Samples were taken at 0, 7, 14, 21 and 28 days to determine the viability of AO-11 cells. The death rate constant per day (*k*), which refers to the decreasing rate of cell viability at each constant temperature, was calculated from the slope of the regression of the log viability versus time, as shown in Eq. (1):^29^

1 $$[{\text{LogN}} = {\text{LogN}}_{0} {-}kt]$$

where N_0_ is the number of initial viable cells, N is the number of viable cells at any time (both expressed as CFU/g carrier) and *t* is the time in days.

The correlation between the temperature (Kelvin) and the *k* values can be described by the Arrhenius Eq. ^30^ as shown in Eq. (2):

2 $$k = k_{0} e^{{\left( { - E_{a} /RT} \right)}}$$

where *R* is the universal gas constant (8.32 J mol^−1^·K), *E*_*a*_ is the apparent activation energy in kilojoules per mol, *T* is the absolute temperature in Kelvin, and *k*_0_ is the pre-exponential constant.

The predicted values of the death rate constant (*k* per day) at the desired storage temperatures over a range that followed the Arrhenius equation were calculated and used to determine the predicted shelf life (*t*) in days, which refers to the period that the immobilized AO-11 product can be stored with retention of 50% degradation activity. The resultant *k* value for each temperature was used in Eq. (1), where N_0_ was 1.18 × 10^10^ CFU g^−1^ carrier and N_t_ was the minimal effective cell concentration (CFU g^−1^ carrier) that had a crude oil removal efficiency greater than 50%. All experiments were performed in triplicate.

### Scanning electron microscopy (SEM) examination of immobilized bacteria

SEM samples of immobilized cells on carriers were fixed in 2.5% (v/v) glutaraldehyde in phosphate buffer for 1–2 h and rinsed with ethanol (30, 50, 70, and 95% (v/v), respectively) for 10 min for each concentration. Then, the samples were washed with 100% ethanol 3 times for 10 min each wash. The carriers were dried and sputter-coated with gold. Observations were performed using SEM (JSM-IT500HR, Jeol Ltd., Tokyo, Japan).

### Statistical analysis

All experiments were conducted in triplicate. The reported data correspond to the arithmetic mean ± standard deviation. Statistical analysis of the experiments  was performed with one-way ANOVA and independent *t* tests followed by Duncan’s multiple range test at a 95% confidence interval using SPSS software version 23.0 for Windows.

## Results and discussion

### Biofilm formation

**Figure 1:**
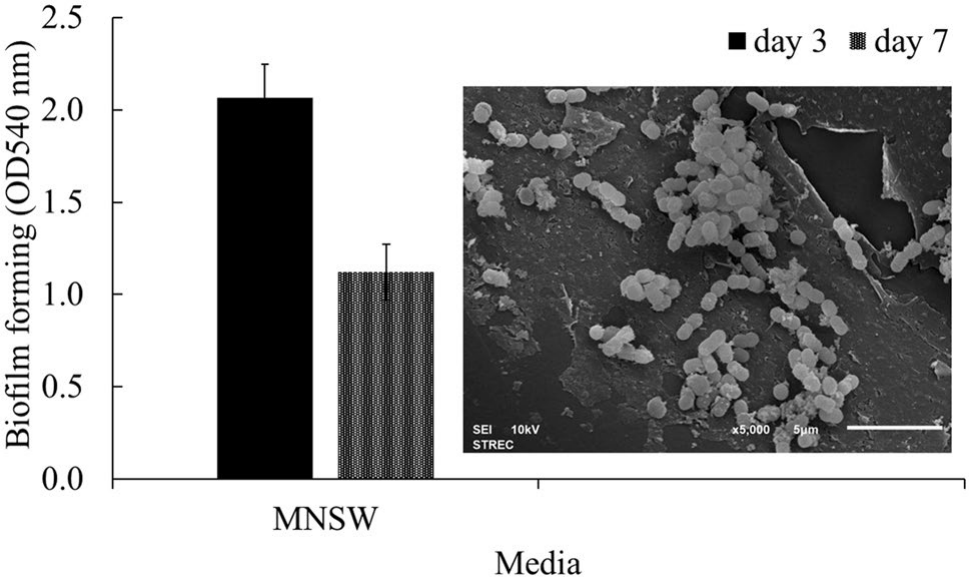
Crystal violet staining assay for testing biofilm formation by *Exiguobacterium* sp. AO-11 in MNSW medium incubated at room temperature for 3 and 7 days, and SEM micrographs of immobilized cells on bio-cord after 3 days of cultivation at room temperature with continuous shaking at 200 rpm. Data correspond to the means of triplicate values, and error bars correspond to the standard deviation.

The production of biofilms represents a primary bacterial strategy for adherence to a surface to create micro-communities for survival^31^, which is useful in protecting cells from adverse conditions and predators while preserving bacterial survival without decreasing bacterial activity^32^. Additionally, the biofilm-mediated bioremediation process offers an effective and low-cost method over free cells^33^. In this study, the biofilm formation potential of *Exiguobacterium* sp. AO-11 was determined prior to the immobilization of bacterial cells on the supporting material in subsequent experiments. The results showed that on day 3, this strain produced moderate biofilms, but there was a significant decline in biofilm amount on day 7 (Fig. 1). As biofilm formation activity could facilitate the adhesion of bacterial cells on the surface of materials, the adherent AO-11 cells on the supporting material (bio-cord) are shown in the SEM micrograph (Fig. 1). The decrease in biofilm-forming capacity on day 7 could be ascribed to the loss of exopolymers, particularly exopolysaccharides, from the biofilm. This may indicate the occurrence of biofilm detachment, probably facilitated by enzymatic degradation^34^. *Exiguobacterium* species have been reported as biofilm producers and were isolated from natural biofilm communities, such as biofilms in Ginger Lake, Antarctica^35^ and shallow lagoons^36^. This finding confirmed that *Exiguobacterium* sp. AO-11 had the potential to produce biofilms for adhesion on the carrier surface. Furthermore, the removal of xenobiotic compounds, such as crude oil^37^ and polycyclic aromatic hydrocarbons (PAHs)^38^, by biofilm-forming strains immobilized on suitable carriers was reported.

### Selection of the carrier for immobilization

The use of appropriate carriers for microbial immobilization is known to reduce competition with indigenous microorganisms and is generally intended to provide a protective microenvironment for microbial inoculants, which results in the retention of their microbial viability and activity^8^. In this study, three types of carriers were considered for the selection of suitable immobilized carriers. First, polyurethane foam is an inert material with good mechanical properties (high resistance and elasticity) and high porosity (approximately 97%) and hence a large adsorption surface, which reduces the problems of oxygen diffusion to aerobic microorganisms^39^. Second, aquaporous gel is a highly hydrophilic polyurethane sponge that provides a hydrophilic surface for chemical-degrading bacteria to attach to and colonize, has high wearability, and is an anti-floating material. The last carrier, bio-cord (polypropylene fine fibers), facilitates immobilization of a large number of microorganisms in the spaces within the carrier and provides suitable conditions and support for microbial growth^40,41^. The results of each carrier presented in Table 1 showed that of the 3 types of carriers, bio-cord was a suitable carrier for preparing immobilized *Exiguobacterium* sp. AO-11. It exhibited the highest viability of immobilized cells at 2.14 ± 0.63 × 10^9^ CFU g^−1^ carrier, which was significantly different from those of other carriers. The retained high biomass of the bacterial population on the supporting material is one of the key characteristics for cell immobilization^5^. Furthermore, the crude oil adsorption capacity and cost of the carrier are attractive choices for selecting an adsorbent material for use to remove oil from an oil spill site^42^. The adsorption capacity of bio-cord (1.16 ± 0.01 g g^−1^ of carrier) was higher than that of aquaporous gel (0.15 ± 0.01 g g^−1^ of carrier), which indicated that bio-cord could not only act as a bacterial carrier but could also adsorb crude oil. In summary, bio-cord is an attractive carrier owing to its retention of high cell viability, low price, and high crude oil adsorption capacity, thus making it a useful and durable carrier for immobilization of *Exiguobacterium* sp. AO-11.

**Table 1:** Price, adsorption capacity, quantity of immobilized cells and characteristics of cells immobilized on carriers under scanning electron microscopy (SEM) separated by the type of carrier including polyurethane foam, aquaporous gel and bio-cord.

Carriers	Cost/kg of substrate (USD)	Oil adsorption capacity (g g^−1^ of carrier)*	Immobilized cells (CFU g^−1^ carrier)*	Surface of each substrate	Immobilized *Exiguobacterium* sp. AO-11
Polyurethane foam	36.67	1.57 ± 0.01^c^	1.73 ± 0.35 × 10^7a^		
Aquaporous gel	1.17	0.15 ± 0.01^a^	1.10 ± 0.66 × 10^8a^		
Bio-cord	11.67	1.16 ± 0.01^b^	2.14 ± 0.63 × 10^9b^		

*Values are expressed as the means ± sd. The values in each column with the same superscript letter are not significantly different by Duncan’s multiple range test (*P* ≤ 0.05).

### Crude oil removal efficiency

The immobilized AO-11 cells showed a crude oil removal efficacy of 68.66 ± 14.27% of the initial oil (0.25% (v/v) of crude oil, approximately 2,000 ppm), while free AO-11 cells achieved 45.1 ± 4.27% removal after 9 days of incubation. Both the adsorption and biodegradation capacities could enhance the oil removal efficacy of immobilized cells^14^. The results implied that the free cell form allowed the crude oil to be easily accessed by microorganisms^5^, whereas immobilization did not inhibit biodegradation by the cells, and the carrier could adsorb crude oil, which resulted in increased bioavailability within cells^43^. However, the crude oil removal efficacy of AO-11 shown in this study was lower than that of the previous report by Muangchinda, et al.^6^. In a previous report, AO-11 showed a high oil removal efficacy of 91.60 ± 1.20% within 10 days when it was cultivated in natural seawater medium (NSM), which consisted of the same composition as MNSW except for the concentration of seawater. NSM contained 20% (v/v) seawater instead of 100% seawater, as in MNSW, indicating that salinity is one of many factors that impacts crude oil removal efficiency. Similarly, Cao, et al.^44^ reported that an increase in salinity affected the behavior of the oil-degrading bacteria *Exiguobacterium* sp. N4 − 1P, and the highest degradation and biofilm formation were observed at a salinity of 15 g L^−1^. Therefore, the effects of various environmental factors, such as the crude oil concentration, pH, salinity, cell starvation, and the presence of oil dispersant on the crude oil removal efficiency of immobilized AO-11 were further investigated.

### Effects of the crude oil concentration, pH and salinity on the crude oil removal efficacy

**Figure 2:**
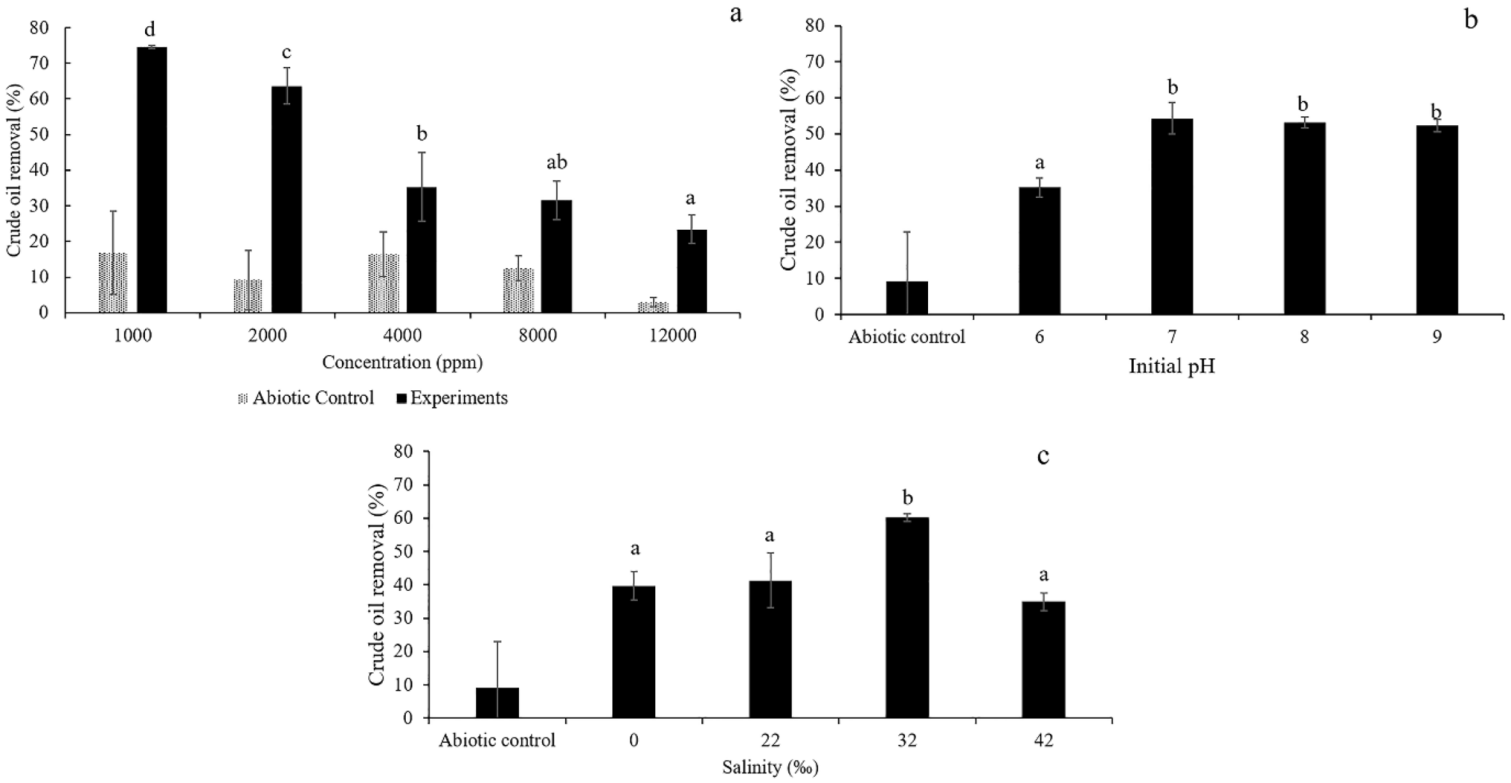
Effects of crude oil concentrations in the range of 1,000–12,000 ppm (**a**), initial pH (**b**) and salinity (**c**) on crude oil removal by immobilized *Exiguobacterium* sp. AO-11 cells were incubated at room temperature with continuous shaking at 200 rpm for 9 days. Data correspond to means of triplicate values, and error bars correspond to the standard deviation. The different letters represent significant differences at *P* ≤ 0.05.

The crude oil removal efficiencies of the immobilized AO-11 cells in the different initial concentrations of crude oil, pH values and salinities were investigated to design the usage scope of the immobilized cells. High or low concentrations of pollutants affect microbial degradation and lead to a decrease in microbial degradation efficiency^7^. The effect of crude oil concentration on the removal efficiency of immobilized AO-11 cells was examined in MNSW (pH 7.0 ± , salinity 32‰) containing 1,000, 2,000, 4,000, 8,000 and 12,000 ppm crude oil. The removal efficiency of immobilized AO-11 cells reached maximum values of 74.2% (196 ± 3 ppm residual crude oil concentration) at an initial oil concentration of 1000 ppm. When the concentration increased to 2,000, 4,000, 8,000 and 12,000 ppm, the removal percentage decreased significantly (crude oil removal percentage (%); residual crude oil (ppm)) (63.6 ± 5.1%; (685 ± 154), 35.3 ± 9.6%; (2,062 ± 302), 31.5 ± 5.33%; (6,319 ± 505) and 23 ± 3.9%; (9,691 ± 535), respectively) (Fig. 2a). The results demonstrate that crude oil degradation is inversely proportional to the concentration of oil^45^, which could be ascribed to the toxicity of hydrocarbons^46^ at such high crude oil concentrations, which, in turn, might have negatively affected the biodegradation activities of the tested bacterial strains^47^.

The effect of the initial pH of the medium on the crude oil removal activity of immobilized AO-11 cells was examined, and the results revealed that crude oil removal at pH 7–9 was not significantly different. The maximum crude oil removal was observed at a pH of 7.0, and higher activity was retained under alkaline conditions (pH = 8.0–9.0) than under acidic conditions (pH = 6.0) (Fig. 2b). This experiment demonstrated that immobilized AO-11 cells were appropriate for the alkaline environment, making them suitable for use in seawater that is typically slightly alkaline^48^, and it seems to be a highly favorable condition for petroleum hydrocarbon degradation^17^.

Salinity is another important factor that may affect oil biodegradation through alteration of the survival and metabolism of bacteria. To investigate the tolerance of immobilized AO-11 cells over a wide salinity range, crude oil removal trials were determined over a salinity range of 0–42‰, which represented different environments as follows: 0‰ represented fresh water, 22‰ represented brackish water, and 32–42‰ represented sea water^49^. The results demonstrated that the maximum crude oil removal efficiency was observed at 32‰ (Fig. 2c). Meanwhile, at other salinity values, the immobilized AO-11 cells also showed an oil removal capacity, but at a lower percentage. This finding was consistent with a report of naturally existing bacteria isolated from seawater whose crude oil degradation efficiency reached a maximum value at a salinity of approximately 30‰^7^. Furthermore, salinity strongly affects the behavior of oil-degrading bacteria and biofilm formation^44^.

The obtained data on the capacity of immobilized AO-11 cells allowed them to be appropriately designed for future use. The comparison of crude oil removal under all conditions rather than as individual experiments will be a fascinating aspect. The combination of factors affecting crude oil degradation can be further examined by response surface methodology (RSM) after significant data on individual factors that affect crude oil degradation are obtained^50^. Further multivariate optimization design might have led to a higher optimal oil removal rate.

### Effect of the starved state of free and immobilized *Exiguobacterium* sp. AO-11 on crude oil removal

**Figure 3:**
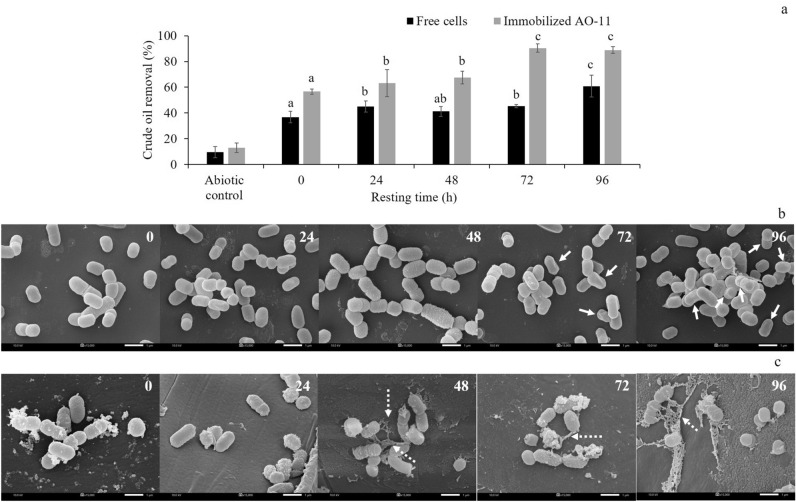
Crude oil removal of free cells and immobilized *Exiguobacterium* sp. AO-11 after starvation for 0, 24, 48, 72 and 96 h in MNSW containing 0.25% (v/v) crude oil incubated at room temperature for 9 days (**a**). SEM micrographs of free cells (**b**) and immobilized AO-11 cells on bio-cord (**c**) after starvation arranged from left to right of the pictures (15,000 ×): 0 h; 24 h; 48 h; 72 h; and 96 h. The white arrows indicate morphological changes and the accumulation of free cells at 72–96 h. The white dashed arrows indicate biofilm produced by *Exiguobacterium* sp. AO-11. Data correspond to the means of triplicate values, and error bars correspond to the standard deviation.

Starved state-induced microorganisms are said to represent the priming step and are ready for nutrient assimilation. Under the starved state of cells, the degradation of nutrients occurred faster than under typical conditions^51^. In this study, it was found that when the resting time was increased, both free and immobilized AO-11 cells significantly increased the crude oil removal efficacy. A resting time of more than 72 h showed an enhanced crude oil removal efficiency of up to 90.48 ± 3.13% by immobilized AO-11 cells. Meanwhile, a resting time of 96 h enhanced crude oil removal by free cells, reaching 60.86 ± 8.54% (Fig. 3a). Interestingly, the starved period of free cells was prolonged compared with immobilized AO-11 cells, suggesting that the aggregation of free cells and appearance of biofilm helped to create bacterial communities for bioremediation, which is a more proficient method than planktonic microorganisms, as the biofilm environment protects them during stress^52^ and from the toxicity of crude oil, resulting in an increased crude oil removal efficiency. Recently, a report by Moreno and Rojo^51^ explained the regulatory mechanisms of the alkane degradation pathway during the starvation state using *Pseudomonas putida* as a model. The alternative sigma factor σ^s^, which is expressed during starvation, allowed a rapid response to the presence of *n*-alkanes by providing the accumulation of transcriptional activators that are essential for the activation of genes for alkane degradation.

The scanning electron micrograph of free cells showed a different cell surface structure during starvation when cells were starved for 72 h, whereas at 96 h, free cells were found to aggregate, and biofilm production occurred (Fig. 3b). In contrast to immobilized AO-11 cells, the cell surface remained the same, while cell aggregation was observed before resting treatment, and biofilm production was increased after an increase in resting time (Fig. 3c). A change in cell morphology due to starvation is often found in the natural environment under nutrient-limiting conditions^53^. This change may be related to low-nutrient conditions and the reduced expression of genes encoding proteins that determine the size and shape of the cells^54^. At the molecular level, the reason for the increase in the crude oil removal activity by *Exiguobacterium* sp. AO-11 after increasing the resting time has not been determined. Further research at the gene regulation level would be helpful for achieving an in-depth understanding of the starvation responses and crude oil removal efficiency in *Exiguobacterium* sp. AO-11.

### Effects of the oil dispersant on survival and crude oil removal

Slickgone NS is a widespread commercial oil dispersant used to reduce the impact of petroleum oil spills^55^. The effect of Slickgone NS at a ratio of 1:25 DOR on the viability and crude oil removal of free and immobilized AO-11 cells was examined. The results showed that the presence of Slickgone NS did not significantly impact the viability of either free or immobilized cells. Furthermore, the addition of Slickgone NS showed positive effects on the crude oil removal efficiency of free cells, as in a previous study^55^. The removal efficiency of free cells increased to 85.7 ± 2.25%, while the high efficacy of oil removal of immobilized *Exiguobacterium* sp. AO-11 was maintained at 89.22 ± 0.89%, as shown in Table 2. It was suggested that Slickgone NS reduces the interfacial tension between oil and water^56^, generating larger numbers of small droplets of oil, which enhances substrate availability.

**Table 2:** Total viability and crude oil removal efficiency of free and immobilized *Exiguobacterium* sp. AO-11 after being cultured with Slickgone NS.

	Total cell count in suspension (CFU mL^−1^)		Total cell count on bio-cord (CFU g of carrier^−1^)		Crude oil removal (%)	
	Control	AO-11 + Slickgone NS	Control	AO-11 + Slickgone NS	Slickgone NS added	Without Slickgone NS
Free cells	6.33 ± 1.45 × 10^7^	6.33 ± 0.67 × 10^7^			85.7 ± 2.25	60.86 ± 8.54
Immobilized AO-11 cells	2.28 ± 1.19 × 10^6^	2.09 ± 0.79 × 10^6^	2.50 ± 0.43 × 10^9^	3.23 ± 4.58 × 10^9^	89.22 ± 0.89	90.48 ± 3.13

### Reusability of immobilized *Exiguobacterium* sp. AO-11

**Figure 4:**
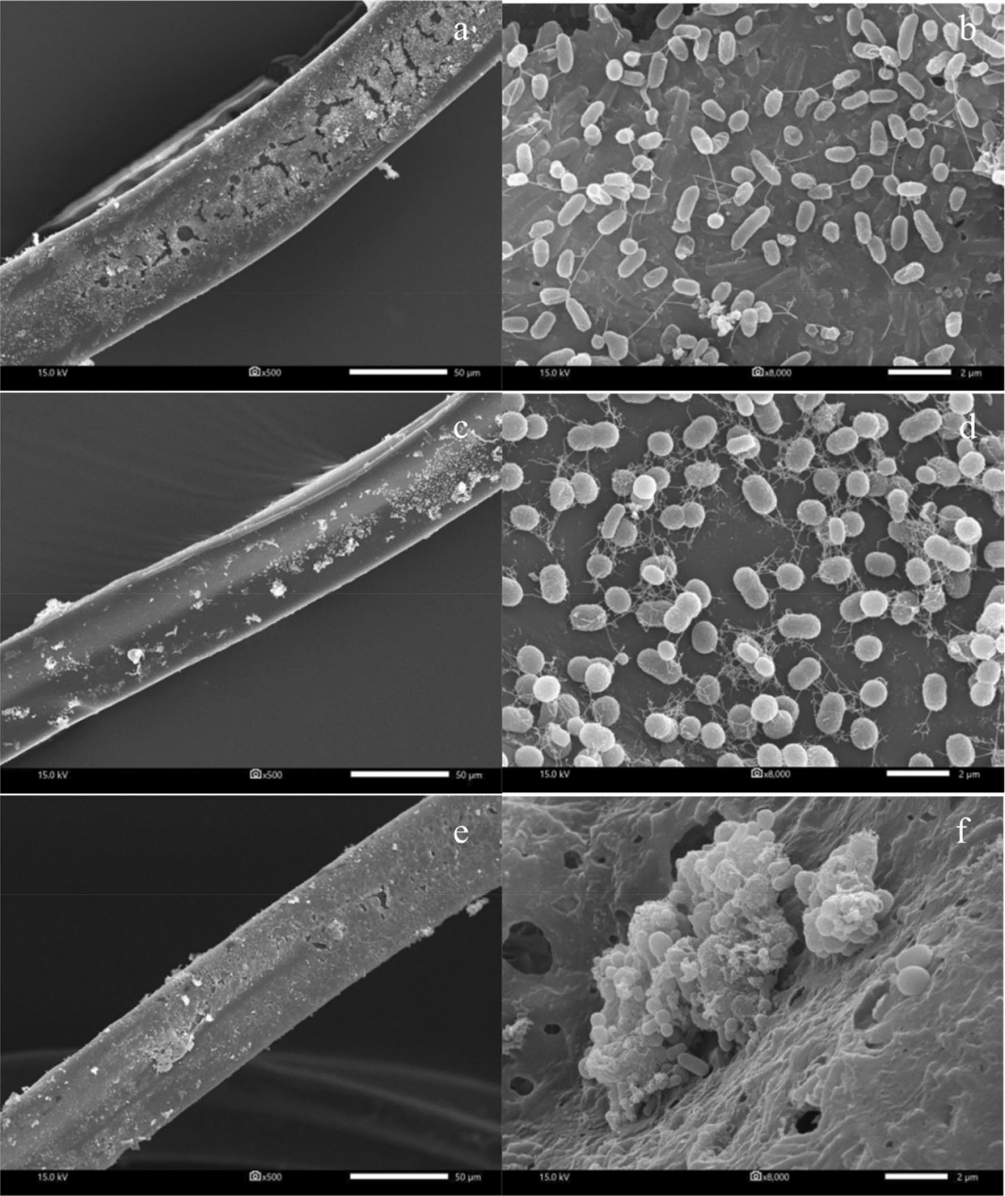
SEM micrograph of the surface of bio-cord colonized with *Exiguobacterium* sp. AO-11 at 500 × (left) and a close-up SEM micrograph of the bio-cord surface with *Exiguobacterium* sp. AO-11 attached at 8,000 × (right) after reuse for 1 (**a**,**b**), 3 (**c**,**d**) and 5 (**e**,**f**) cycles.

Reusability is another advantage of immobilized cells by reducing waste while saving time and cultivation costs^11^. The removal efficacies of immobilized AO-11 cells were 88.24 ± 2.05, 83.69 ± 11.96 and 86.51 ± 0.55% after the first, third and fifth cycles of use, respectively. This finding suggests that the crude oil removal activity of immobilized AO-11 cells could be maintained, and they could be reused without any loss of activity. The reusability efficiency of immobilized cells has been reported previously, and immobilized cells can maintain removal activity, making them suitable for bioremediation^9^. The scanning electron micrograph of immobilized AO-11 cells showed increased biofilm formation and retention of cell viability until the last cycle of use (Fig. 4).

### Shelf life evaluation of immobilized AO-11 product

#### Selection of storage conditions

**Figure 5:**
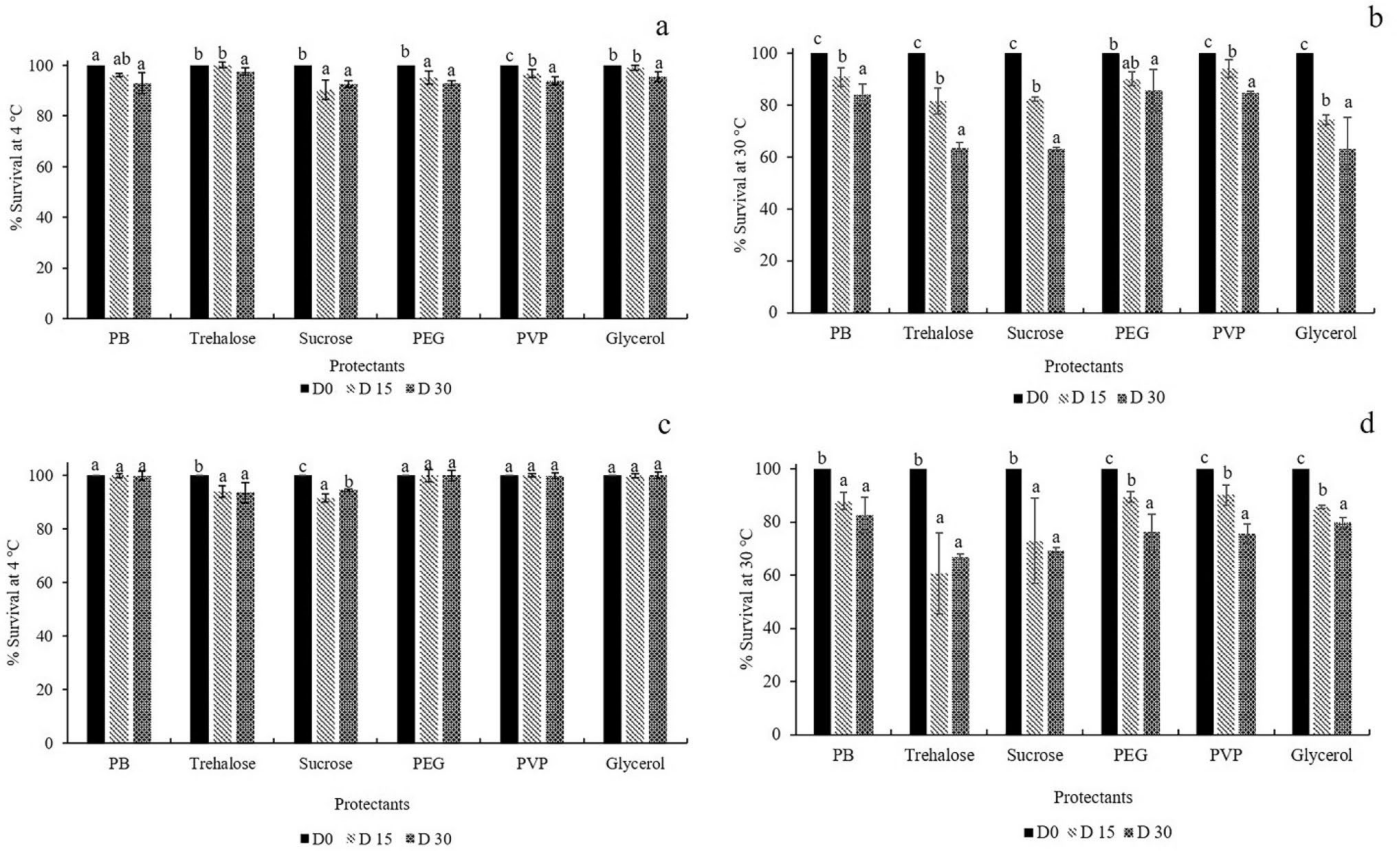
Percentages of immobilized *Exiguobacterium* sp. AO-11 cell survival after storage for 15 and 30 days with various protectants, including PB, trehalose, sucrose, PEG, PVP, and glycerol, at 4 °C and 30 °C under non-vacuum (**a**,**b**) and vacuum (**c**,**d**) conditions, respectively. Data correspond to the means of triplicate values, and error bars correspond to the standard deviation.

The storage conditions of immobilized AO-11 cells were examined in bags containing phosphate buffer and different types of protective agents. The immobilized cells were stored at 4 °C and 30 °C under vacuum and non-vacuum conditions. Evaluations of the survival percentage of immobilized products (Fig. 5) revealed that storage of the immobilized products at 4 °C gave a survival percentage above 92% in all conditions. The results implied that preservation at low temperature may slow down or inhibit microbial metabolism, which led to high survival^57^. Meanwhile, storage at 30 °C in phosphate buffer without the addition of protective agent showed high survival percentages of 82.61 ± 6.67 and 84.34 ± 3.39% after 30 days of storage under vacuum and non-vacuum conditions, respectively. These results suggest that the metabolism of cells was reduced under carbon starving conditions, which allows them to survive after a month of storage. Our results were in accordance with previous studies, in which the survival percentages of *Pseudoxanthomanas* sp. RN402 and the liquid formulation of *Exiguobacterium* sp. AO-11 preserved in phosphate buffer were 94 ± 1.5% and 73.8 ± 2.2%, respectively, after storage for 30 days at 30 °C^6,28^. Hence, no addition of protective agent in phosphate buffer and no preparation under vacuum conditions were required for the storage of immobilized AO-11 cells, which has advantages with regards to the ease of preparation and cost-effectiveness.

#### Effect of temperature

The percent survival of immobilized AO-11 cells was an important index to predict the shelf life of the product. The viability of immobilized AO-11 cells decreased continuously at high storage temperatures, in contrast to storage at low temperatures, which resulted in a slight decrease in cell viability. This finding demonstrated that immobilized AO-11 cells were sensitive to elevated temperatures, which was one of the most important abiotic factors affecting shelf life^19^. The analysis data indicated that the survival graph of immobilized AO-11 cells fit with the first-order equation, in which the slope of the equation refers to the death rate constant (*k*). There was a directly proportional relationship between the mortality of the immobilized AO-11 cell concentration and the storage temperature over a range of 5–40 °C, as shown in Table 3.

**Table 3:** Death rate constant (*k*) of immobilized *Exiguobacterium* sp. AO-11.

Sample	Death rate constant (*k*) (d^−1^)*			
	5 °C	15 °C	30 °C	40 °C
Immobilized *Exiguobacterium* sp. AO-11	0.0143	0.0219	0.0533	0.2027

*Death rate constant (*k*), which refers to the decreasing rate of cell viability at each constant temperature.

#### Prediction of the storage stability

**Figure 6:**
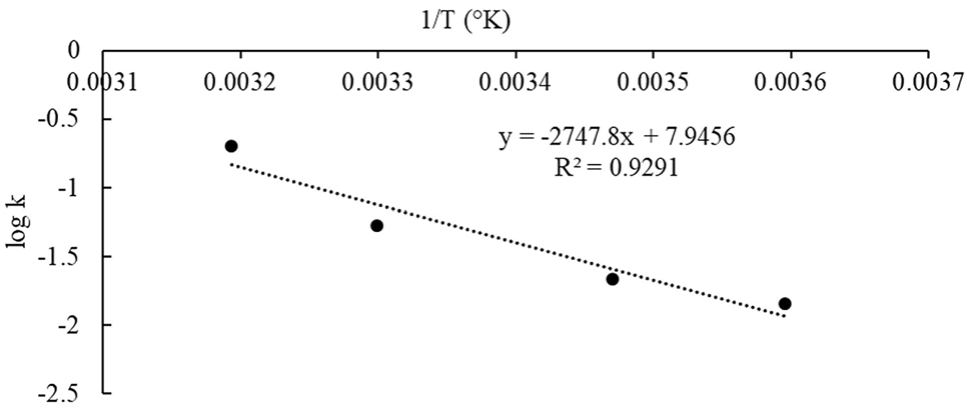
Arrhenius plot of thermal degradation by immobilized *Exiguobacterium* sp. AO-11 under non-vacuum condition.

Accelerated storage testing is an essential tool for predicting the storage time limits at which the desired cell concentration can be maintained at each storage temperature^58^. The obtained data can be used to design suitable storage conditions for the marketplace. The effect of the storage temperature (5–40 °C) on the death rate constant per day (*k*) under non-vacuum storage conditions followed the predicted values from the Arrhenius equation. The *k* value at the target storage temperatures of 5–40 °C was obtained from the equations (Fig. 6). The variation in the *k* value was in accordance with the change in temperature because it is the factor that can induce metabolism in cells^59^. Therefore, the stability prediction models at the desired temperatures under non-vacuum conditions were obtained by calculating the *k* values at the desired temperatures with the Arrhenius equations (Fig. 6), and the regression equation was obtained as Eq. (3):

3 $$\log k = - 2747.8\left( {\frac{1}{T}} \right) + 7.9456$$

Therefore, the prediction values of stability at the desired temperature were obtained by replacing the values of N_0_ (1.18 × 10^10^ CFU g^−1^carrier), N (1.0 × 10^7^ CFU g^−1^carrier), and *k* in Eq. (1). The predicted storage time at ambient temperature in Thailand when kept in a non-vacuum bag, as calculated from the equation, was approximately 39 days. To increase the storage time, increasing the initial number of immobilized cells at the beginning of the immobilization step was important for extending the shelf life^58^, and storage at 4 °C was recommended to keep the immobilized cells stable for a longer period^60^. In this study, validation of the Arrhenius equation was performed, and the predicted viabilities calculated from the equation at storage temperatures of 30 and 37 °C for 15 days were 2.40 × 10^8^ and 4.91 × 10^7^ CFU g^−1^ carrier, respectively, which were close to those of the validation experiments using immobilized AO-11 cells (4.20 ± 1.0 × 10^8^ and 1.31 ± 0.19 × 10^7^ CFU g^−1^ carrier, respectively), confirming that the model can be used to predict the stability of immobilized AO-11 cells.

## Conclusion

This work successfully demonstrated crude oil removal capacity under contaminated marine conditions by immobilized nonpathogenic *Exiguobacterium* sp. AO-11. Notably, crude oil removal activity was dramatically enhanced after cells were starved for 72 h. Physical factors such as pH, crude oil concentration, and salinity play significant roles in the biodegradation of crude oil. Additionally, immobilized cells were used in combination with chemical dispersant, and cells were reused for up to five cycles without any loss in activity. Furthermore, these immobilized cells could be stored for at least one month at room temperature in a plastic bag with sustained viability and activity. The findings of this work are expected to serve as a useful guide for the future development of bioremediation products with long shelf lives for commercial purposes. Furthermore, studying gene regulation during cell starvation would be highly beneficial in gaining a fundamental understanding for future research.
